# Nanotechnology in the Treatment of Diabetic Complications: A Comprehensive Narrative Review

**DOI:** 10.1155/2021/6612063

**Published:** 2021-04-30

**Authors:** Yujing He, Abdulrahman Al-Mureish, Na Wu

**Affiliations:** ^1^Department of Endocrinology, Shengjing Hospital of China Medical University, Shenyang 110004, China; ^2^Clinical Skills Practice Teaching Center, Shengjing Hospital of China Medical University, Shenyang 110004, China

## Abstract

In today's society, the prevention and treatment of diabetes mellitus and its subsequent complications have brought trouble to human beings. Complications caused by diabetes bring not only physical and mental pain to patients but also a heavy economic burden to families. And once diabetic complications occur, they are often irreversible and very difficult. At present, some studies suggest that nanotechnology can treat some diabetic complications. This paper reviews the application of nanotechnology in the repair of diabetic segmental bone injury, the healing of diabetic skin ulcers, the therapeutic effect, and improvement strategies and deficiencies of nanotechnology in diabetic complications.

## 1. Introduction

Diabetes is a common disease in life. Its long-term hyperglycemia will cause damage to the various tissues and organs of the body and may lead to complications. These complications will have a significant impact on patients, with diabetic complications involving acute metabolic disorders such as DKA and hyperosmolar hyperglycemia syndrome; there are some common chronic complications of diabetes such as diabetic nephropathy, diabetic retinopathy, diabetic cardiomyopathy, peripheral neuropathy, diabetic foot, segmental bone injury, and other complications [1]. Diabetic complications are often irreversible, causing severe burdens and injury to patients both economically and physically.

Nanotechnology, as a newly developed discipline, has been innovative and applied in the fields of materials science, environmental science, biological science, medicine, chemistry, and so on. In recent years, more and more research has applied nanotechnology to the study of diabetic complications, mainly including treatment and strategy. This paper is going to review this part.

## 2. Introduction to Nanotechnology

Nanotechnology is a new and important technology in the 21st century. It is a kind of science and technology that uses single atom and molecule to make materials. It studies the properties and applications of materials with structure size ranging from 0.1 to 100 nm. Through the arrangement and combination of various particles, a new arrangement of matter is created. With the rapid development of nanotechnology, it has been widely used in medicine, materials science, electronic industry, energy industry, and other disciplines and fields [2, 3]. Nanomaterials have strong activity, and there are different interactions between different nanounits. This characteristic makes nanomaterials have unique effects, such as quantum effect of increasing surface energy, size effect caused by large specific surface area, small crystal structure and small size, and interface effect due to the rapid increase of surface atomic ratio [4–6]. It is due to the in-depth study of the properties of nanomaterials, combined with the continuous improvement of molecular biology technology, new material technology, and other disciplines and technologies; modern medical research and nanotechnology have been organically combined, making nanotechnology broaden a new direction; medical research has also gone deep into a level, especially in the diagnosis and treatment of diseases [7]. Due to its small molecular size and multiple functions, nanotechnology is also added to the research of modern drugs, which is conducive to solve the barrier problem encountered in conventional treatment [8, 9]. Nanodrugs can be targeted to transport drugs and reduce or avoid the side effects of drugs. At the same time, they have the property of sustained release, which can extend the action time of drugs, improve the stability of drug action, facilitate drug storage, and establish some new drug delivery routes. These effects promote the development of drugs in the direction of low dose, low side effects, simplicity, and convenience. Nanodrugs have the properties of small volume and high drug loading function. They are easy to penetrate the blood vessels but do not cause vascular endothelial damage. They can protect the drugs from the decomposition of enzymes in the receptor and make the local drug concentration higher than other methods of administration, so it can improve the efficacy of drugs and reduce or even avoid the side effects [10]. Compared with the traditional drug delivery system, nanotechnology has been widely used in the preparation of new drugs [11, 12]. The main forms are nanoparticles (NP), nanoemulsion, nanomaterials, and nanodrug crystallization. NP-related research has become a hot spot in the development of nanotechnology. In order to play a role, nanocarrier drugs require appropriate particle size, low toxicity, biodegradability, and good biocompatibility; can accurately reach the lesion location; ensure the concentration; produce therapeutic effect; and do not cause damage to normal cells [13, 14]. At present, the main nanotargeting carriers include ordinary nanocarriers, physical targeting carriers, and controlled release nanocarriers [15–17]. A common nanocarrier, also known as nanostructured liposome (NLCs), can be used to change the drug delivery properties of nanoparticles, such as drug delivery and drug delivery [18–20]. These carriers not only can carry a variety of drugs but also have been used in clinical practice, such as anticancer drug carrier [21], antibacterial drug carrier [22], hormone drug carrier [23], and antiparasitic drug carrier. And there are many ways of administration, such as intravenous injection, intramuscular and subcutaneous injection, oral administration, and nasal administration [24]. Controlled release preparation is a kind of dosage form that can release drugs at regular, quantitative, and uniform speed through controlled release, so that the blood drug concentration is constant and there is no “peak valley” phenomenon, so as to better play the curative effect, improve the medication compliance, and reduce the side effects. NP has ultrasmall volume, can pass through the tissue gap and be absorbed by cells, can pass through the thinnest capillaries of the human body, and can pass through the blood-brain barrier [25]. Controlled release nanocarriers can use NP to encapsulate or adsorb active components to reach the target site, and then slow release can be controlled to achieve therapeutic effect. At present, it has been widely used in various fields, such as doxorubicin-loaded newly modified chitosan nanoparticle-controlled release carrier [26], and insulin-controlled release system in diabetes treatment [27]. A physicochemical targeting carrier refers to the preparation that uses some physical and chemical methods to make the targeted preparation exert its efficacy in specific parts, such as magnetic targeting, embolic targeting, thermosensitive targeting, and pH-sensitive targeting. Because of the changes of pH, temperature, and other factors in the lesions under pathological conditions in vivo or through the effect of external magnetic field, targeted localization can be carried out through the sensitivity of nanocarriers, and drugs can be accurately accumulated and released in the lesions, such as albumin nanoparticles [28], and polylactic acid nanoparticles [29].

Nanoemulsion is a nonequilibrium system with an average particle size of 1-100 nm in the proper proportion of oil phase, water phase, and surfactant. It has low viscosity, high efficiency, high permeability, low preparation difficulty, and high safety. It can improve the bioavailability. Because of its special particle size, it also has slow release and targeting effects [30].

At the same time, according to the characteristics of the drug and the different emulsion, it has different ways of administration: transdermal administration, oral administration, mucosal administration, injection administration, etc. [31].

The nanomaterials are hydrogel, a three-dimensional network system with a particle size less than 200 nm [32]. With the same hydrogel, high water content, and swelling characteristics, nanomaterials have a three-dimensional network structure, which can encapsulate active components to protect the active components from environmental damage [33]. Similar to nanoparticles, nanomaterial has smaller particle size, superior properties of nanomaterials, and large specific surface area, which is beneficial to improve the bioavailability of drugs. It can be used for the delivery of chemotherapy drugs [34, 35], protein drugs [36], and gene [37].

In recent years, nanotechnology has played an important role in the diagnosis and treatment of diabetes [38, 39]. It also has good performance in the diagnosis and treatment of diabetic complications [40]. The application of nanotechnology in diabetic complications is described in detail below.

## 3. Common Complications of Diabetes Mellitus and Application of Nanotechnology

In recent years, with the continuous improvement of people's living standards and quality, people's dietary structure and daily habits have undergone great changes, which also makes the incidence of diabetes increased. In recent years, the number of diabetic patients in the world has increased year by year and the global prevalence rate of diabetes 9.3% in 2019 [41]. Diabetes is a metabolic disease characterized by chronic persistent hyperglycemia due to a variety of reasons. Once the disease has occurred, it is difficult to cure it completely. A large number of clinical studies have shown that long-term hyperglycemia of the body may lead to a variety of concurrent diseases. Diabetes can cause great pressure on major organs of the body (cardio cerebrovascular, liver, kidney, etc.) and cause organ damage. The follow-up complications are also very serious and may even affect the lives of patients [42]. And all of these have been made a summary in Table 1.

### 3.1. Diabetic Nephropathy

Diabetic nephropathy (DN) is one of the main causes of nephropathy. At present, there is no effective means to prevent the occurrence and development of DN, and the prevention and treatment of DN have not achieved satisfactory results. In recent years, nanotechnology has been added to the treatment of DN, which can improve the efficacy of drugs and the prognosis of patients with diabetes, thus effectively reducing the pain and economic pressure of patients. The pathogenesis of DN is complex, including the mutation of genetic factors; the imbalance of glucose metabolism leads to the activation of multiple endocrine pathways; there is a production of a large number of endothelial nitric oxide synthase and advanced glycation end products (ages); the inhibition of the formation of nitric oxide is also an inducing condition of the disease, including insulin inhibition, inflammatory reactions, and renal hemodynamic changes. These conditions jointly promote the occurrence and development of the DN Exhibition [43, 44]. Studies have shown that advanced glycation end products (RAGE) and various binding ligands are the key molecules in DN [44, 45].

Poly(lactic-co-glycolic acid) (PLGA) is a kind of synthetic polymer material. Yang [46] developed and optimized the nanoformula of crocin (CT-PLGA-NP), a therapeutic drug for diabetic nephropathy induced by streptozotocin (STZ). In the experiment, CT-PLGA-NPs showed the accumulation of the drug in the kidney and liver of diabetic rats, providing renal antifibrosis and anti-inflammatory effects. CT-PLGA-NP treatment significantly decreased the production and expression of renal fibrosis factors (TGF-*β*1 and fibronectin) and inflammatory cytokines including MCP-1 and TNF-*α* and significantly reduced the activation of NF-*κ*B expression and PKC activity. According to the existing research results, we can conclude that CT-PLGA-NPs can reduce diabetic nephropathy through antifibrosis and anti-inflammatory effects.

Ahangarpour et al. [47] evaluated the effects of myricetin solid lipid nanoparticles (SLN) on streptozotocin nicotinamide- (STZ-NA-) induced DN in mice. Myricetin and its SLN administration improved DN changes by reducing oxidative stress and increasing antioxidant enzyme levels, and these effects were more obvious in SLN-treated mice.

The nanoliposomes containing Eprosartan mesylate were prepared by Ahad et al. [48], in STZ-induced diabetic nephropathy in Wistar rats; serum creatinine, urea, lactate dehydrogenase, total albumin, and malondialdehyde decreased significantly, which indicated that Eprosartan mesylate-loaded nanoliposomes showed renal protection.

### 3.2. Diabetic Retinopathy

Diabetic retinopathy (DR), with an increasing number of diabetic patients, has become the first cause of blindness in 16-64 years of age [44]. The predisposing factors of DR are very complex, such as hypertension, genetic factors, and onset time of diabetes. The pathological changes usually refer to retinal edema caused by retinal capillary endothelial damage, basement membrane thickening, microvascular blockage, and blood retinal barrier function damage, resulting in retinal edema and neovascularization [44, 49].

Nanotechnology is widely used in the treatment of DR and has entered a new stage and has been applied in clinical practice. Some researchers have developed silicate (SI) nanoparticles and tested their antiangiogenesis effects on retinal neovascularization. Histological analysis showed that silicon nanoparticles had no toxic effect on retinal tissue and could inhibit the formation of retinal neovascularization. Therefore, silicon nanoparticles can effectively treat retinal neovascularization induced by vascular endothelial growth factor [50, 51]. Kim and his colleagues reported another study involving gold nanoparticles, which showed that gold nanoparticles inhibit retinal neovascularization. The results showed that gold nanoparticles effectively inhibited the proliferation and migration of retinal microvascular endothelial cells and the formation of capillary like network induced by vascular endothelial growth factor. In addition, the safety of gold nanoparticles is also guaranteed and will not affect the activity of microvascular endothelial cells and cause retinal toxicity [50, 52]. Other types of nanoparticles reported by Jo et al. are titanium dioxide (TiO_2_) nanoparticles. It can effectively inhibit angiogenesis in vitro and has no toxic effect on the retina. Intravitreal injection of particles also inhibited neovascularization. This study shows that TiO_2_ nanoparticles can be used in the treatment of retinopathy mouse models at the concentration level [50, 53]. A study by Gurunathan et al. showed that silver nanoparticles could inhibit VEGF induced angiogenesis in mice [50, 54].

### 3.3. Diabetic Cardiomyopathy

Diabetic cardiomyopathy (DCM) is a myocardial disease that occurs in patients with diabetes and cannot be explained by other cardiac diseases. Some studies have shown that oxidative stress injury to myocardial cells caused by diabetes mellitus is an important factor leading to a series of complications such as DCM, which will eventually lead to myocardial contractile and diastolic dysfunction [55]. Myocardial fibrosis and apoptosis are common pathological features of DCM, which are important reasons for subsequent cardiac functional injury [56]. Microvascular disease is another main pathological feature of DCM. Myocardial microvascular disease caused by diabetes mellitus is closely related to the decline of left ventricular function [57].

In the nanotechnology applied in DCM, PSS-loaded nanoparticles were prepared using poly(lactic-co-glycolic acid) (PLGA) as a drug carrier and modified double emulsion solvent evaporation method. Yu et al. studied the effect of PSS-NP on vascular endothelial function in DCM rats. The results showed that PSS-NP could significantly improve ventricular wall motion and cardiac systolic and diastolic functions in DCM rats. It can regulate the ultrastructure of myocardial microvascular endothelial cells in DCM rat heart, thus slowing the further development of vascular endothelial dysfunction. The concentration of nitric oxide synthase (eNOS) and vascular endothelial growth factor A (VEGFA) in serum was significantly increased, further alleviating vascular endothelial injury in DCM rats [58].

Studies have explored the therapeutic effect of acidic fibroblast growth factor-l (FGFl) nanoliposomes combined with ultrasound targeted microbubble blasting (UTMD) for DCM. This study evaluated the therapeutic effect of FGFl nanoliposomes combined with UTMD by detecting cardiac function indexes. The results showed that the left ventricular systolic pressure (LVESP), left ventricular end diastolic pressure (LVEDP), and maximum rate of left ventricular development pressure (LV ± DP/dtmax) in the FGFl-combined treatment group were significantly better than those in other treatment groups and DCM groups. The results suggest that FGFl can improve myocardial systolic and diastolic function in DCM rats. Collagen volume fraction (CVF) and apoptotic index of cardiomyocytes in each group were measured. The CVF and apoptosis index of cardiomyocytes in the FGFl-combined treatment group were found to be significantly lower than those in other treatment groups and DCM groups. It is suggested that DCM combined with fluorescence light microscopy (FLM) can improve cardiac function in rats. However, the MVD of rats in the FGFl-combined treatment group was significantly higher than that in the DCM group and other treatment groups, suggesting that FGFl-combined treatment can improve the myocardial microvascular lesions of DCM rats, which may increase myocardial blood flow and reduce oxidative stress injury in DCM rats. Therefore, FGFl nanoliposomes combined with UTMD can improve myocardial pathological and functional changes induced by diabetes [59].

It is not difficult to see that nanotechnology has performed well in animal experiments for the treatment of DCM. However, it is worth considering whether it can have good clinical effects as animal experiments and whether the biocompatibility, biodegradability, and drug release timing of the nanodrugs mentioned above are good.

### 3.4. Peripheral Neuropathy

Diabetic peripheral neuropathy (DNP) causes a high incidence rate of diabetic complications, which is a chronic disease and a key cause of foot ulceration and amputation. According to the relevant data statistics [44, 60], for many diabetic patients, DPN often appears along with other complications. At this stage of clinical investigation, it is found that there are many factors inducing DPN, such as microvascular damage and glucose metabolism disorder. At the same time, immune decline, vitamin, and nerve growth factor deficiency and Schwann cell theory are also included.

Satellite glial cells (SGCs) encapsulate the neuronal bodies in the dorsal root ganglion (DRG). Purinergic 2 (P2) Y12 receptor was expressed on SGC in DRG. The activation of SGC plays an important role in the pathogenesis of DNP. Curcumin has anti-inflammatory and antioxidant properties. Due to poor metabolic stability and low bioavailability of curcumin in vivo, encapsulated curcumin nanoparticles were used to improve its targeting and bioavailability. Some scholars have studied the two of them. Curcumin coated with nanoparticles can reduce the upregulation of P2Y12 receptor on SGC in DRG and reduce the mechanical and thermal hyperalgesia of DM rats [61].

At the same time, the effect of nanocurcumin on the severity of sensorimotor polyneuropathy (DSPN) in patients with type 2 diabetes mellitus (T2DM) has been studied. The results show that short-term nanocurcumin supplementation can improve and reduce the severity of DSPN in T2DM patients. In addition, serum FBS and HbA1c levels have decreased significantly. This observation provides a window of opportunity to improve DSPN by controlling hyperglycemia in T2DM patients [62].

Nano-mir-146a-5p improved the velocity of nerve conduction and alleviated morphological injury and demyelination of the sciatic nerve in DPN rats. Nano-mir-146a-5p inhibited the expression of inflammatory cytokines, caspase-3, and cleaved caspase-3 in the sciatic nerve. In addition, mir-146a-5p nanoparticles can promote the expression of myelin basic protein. These results indicate that mir-146a-5p has a protective effect on the peripheral nerve in the DPN rat model, which may occur by regulating inflammatory reactions and apoptosis [63].

### 3.5. Diabetic Foot

Diabetic foot refers to foot ulcer, infection, and deep tissue destruction associated with abnormal distal nerves of lower limbs and different degrees of peripheral vascular disease. It is often a combination of multiple risk factors that eventually lead to diabetic foot. Diabetic foot ulcer (DFUs) is one of the major and serious complications of diabetic patients, which can lead to lower limb amputation or even death, causing physiological pain to patients and also economic burden. The principle of diabetic foot treatment is to create a clean and suitable microenvironment for the local wound of the foot and to promote its repair as soon as possible. Wound healing is a very complex process, which needs to be carried out in order. Therefore, the ideal wound dressing should be nonallergic and nontoxic, can keep the wound environment moist, allows gas exchange, protects the wound from microbial damage, and absorbs wound exudate [64].

Nanotechnology has been widely used in the research of wound dressing for diabetes, such as chitosan, gold, silver, and curcumin. Among these nanomaterials, there is increasing research on structural similarity, biocompatibility, and biodegradability between biopolymers and typical skin. Metal nanoparticles, such as silver and gold, are also good choices for wound dressings due to their antibacterial activity and low toxicity [65].

PLGA has many advantages, such as good biocompatibility, biodegradability, and mechanical strength. Due to its correlation with growth factors, PLGA has become one of the most studied polymers in diabetic wounds. In one study, PLGA microspheres loaded with recombinant human EGF nanoparticles (NPS) were prepared to improve the biological half-life of PLGA microspheres loaded with recombinant human EGF (rhEGF) and to keep growth factors in contact with wound surface. RhEGF was embedded in the wound surface of diabetic rats and released for 24 hours. Proliferation of fibroblasts was the fastest, and the cure rate was the fastest in rhEGF NP group [66]. In addition, rhEGF-loaded PLGA alginate microspheres were developed to reduce the wound area of diabetic rats and improve the reepithelization of full-thickness wounds in diabetic rats [67].

The mixture of antioxidant EGCG, gold nanoparticles (AuNP), and active oxygen scavenger *α*-lipoic acid (ALA) extracted from green tea can reduce the expression of RAGE in fibroblasts (Hs68) and promote wound healing in diabetic mice. In addition, the level of VEGF in the treatment group was higher compared with the control group, suggesting that angiogenesis was improved [63, 68].

Curcumin is a natural product that has been widely used in recent years. It has been reported to have a variety of properties, including anti-inflammatory, antioxidant, anticancer, and antibacterial activity. Recent studies have also shown antidiabetes and promoting wound healing [69]. Curcumin nanoparticle (CNP) gelatin microspheres (GMs) were loaded into hydrogels, and CNP GM/hydrogel improved wound healing, collagen formation, and neovascularization [70]. In addition, silk fibroin hydrogel was prepared using L-carnosine (L-car) as a metal chelating dipeptide and curcumin. The dressing showed better wound contraction, improved reepithelialization, and formed a fresh thick epidermis [71].

Gao et al. [72] successfully prepared the inner loaded DMOG composite nanofiber membrane. The in vitro experimental results showed that the composite nanofiber could promote the expression of angiogenesis, wound repair, and other related genes. The results of in vivo experiments show that the composite nanofibers can promote the process of wound healing and promote the reepithelialization and vascularization of the wound, which shows a good wound repair effect. The results show that the nanofiber membrane has good mechanical properties; its three-dimensional morphology is similar to that of natural extracellular matrix and has good drug-controlled release effect.

Han and others [73] tested the effect of nanoceria on wound healing of diabetic rats. The results of HE and the wound healing rate showed that nanoceria significantly improved the wound healing of rats. The mechanism may be to regulate the metabolism of sugar, lipid, and amino acid by improving the metabolic network of rat wound and upregulate the anti-inflammatory and antioxidation stress signaling pathway, so as to accelerate wound healing.

In the field of wound dressing, the application of nanotechnology is also very gratifying. It not only uses a lot of new synthetic molecular materials but also combines them with natural products. However, the permeability, air permeability, and clinical effect compared to other dressings still need to be compared.

### 3.6. Segmental Bone Injury

Segmental bone defect is very likely to occur after a comminuted fracture caused by trauma. When a bone defect reaches a certain degree, no bone healing can be produced. However, due to the toxicity of hyperglycemia and microvascular complications, bone formation decreases, bone resorption increases, bone turnover is accelerated, and bone mineral loss leads to osteoporosis. Diabetic patients are therefore more likely to have comminuted fracture and segmental bone defect. Its surgical repair needs more filling materials, and postoperative healing is more difficult, which has been a difficult problem for clinicians.

The traditional repair method of segmental bone defect is to fill the defect area with autograft or allogeneic bone transplantation and bone replacement materials. Among all bone transplantation materials, the source of autogenous bone is very limited, the duration of surgery is long, and the number of patient suffering is high; the use of allogeneic bone is easy to produce a rejection reaction and increases the chance of incision infection, and the implanted bone is easy to be absorbed, which leads to failure of operation. Many bone substitute materials (such as hydroxyapatite) have some physical and chemical properties which affect the combination of bone tissue and are not ideal. NHAC is a kind of nanosized bone frame biomaterial based on the bionic principle. Its composition and structure are similar to that of autogenous bone. NHAC is also an ideal repair material for diabetic comminuted fracture and segmental bone defect. It can also solve the shortage of the source of bone extraction, avoid the pain of autogenous bone extraction, shorten the operation time, and mix with autogenous bone particles. It also promotes the healing of fractures [74].

Some scholars have found that the addition of adiponectin nanomicrospheres can promote the adhesion and proliferation of osteoblasts on the surface of titanium implants under type 2 diabetes mellitus, improve the alkaline phosphatase (ALP) activity of osteoblasts, induce more collagen secretion and extracellular matrix mineralization, and promote the expression of osteogenic related genes and their corresponding proteins [75].

### 3.7. Diabetic Macroangiopathy

Diabetic patients are accompanied by hyperglycemia and insulin resistance, which may lead to the formation of atherosclerosis, causing the progression of diabetic vascular diseases. Patients with type 2 diabetes are particularly at risk of vascular injury. In many patients with diabetes, their cholesterol and triglycerides reach dangerously high levels and accumulate in the lumens of their vascular system [76].

As we all know, oxidative stress is the main cause of cardiovascular disease [77]. The risk indexes of coronary heart disease (CHD) include the increase of TC, LDL-C, and TC/HDL-C and the decrease of HDL-C. Therefore, in the past 30 years, the role of selenium in the prevention of cardiovascular disease has received special attention due to its antioxidant activity. Nanoselenium can be used as an antioxidant to reduce the toxic risk of selenium, because the glutathione S-transferase (GST) induction of se is the key mechanism of this chemopreventive effect. Selenium nanoparticles with different sizes (5-200 nm) have the same ability to induce selenoenzyme in cell culture and mice [78]. In the experiments of Liu [79] and others, through the experimental study of different concentrations of nanoselenium treatment, it is concluded that nanoselenium has a certain protective effect on the oxidative damage of cells caused by hydrogen peroxide. We also studied the effect of selenium enriched tea polysaccharide on diabetic cardiovascular disease. We concluded that medium dose selenium-enriched tea polysaccharide can significantly reduce the levels of TG, TC, and LDL-C in diabetic mice, significantly increase the level of HDL-C in diabetic mice, improve dyslipidemia, and inhibit diabetic cardiovascular damage through antioxidant effect.

### 3.8. Other Complications

Oxidative stress caused by diabetes can lead to decreased fertility in diabetic men [80]. The low fertility rate in men with diabetes is very high, because diabetes can have adverse effects on sperm performance, motility, and quality [81–83] and can also damage testosterone synthesis [84]. Some experiments have shown that ZnO nanoparticles can increase the number and activity of sperm and the level of serum testosterone in diabetic rats and restore the structure of spermatogenic epithelium and joints. Immunohistochemical analysis also showed that ZnO-NP could restore the number of primary spermatocytes, spermatogonia, and Sertoli cells with supporting and nutritional functions [85].

## 4. Conclusion

As a new technology, nanotechnology is the most promising scientific field of this century. With the development of nanotechnology, more and more new nanomaterials will be developed and applied in medical treatment, which will promote the development of modern medicine, bring forward new ideas, and make new contributions to the prevention and treatment of diseases.

Under the condition of modern medical treatment, the incidence rate of diabetes is still rising. The complications of diabetes are more and more harmful to patients. Effective treatment measures are of great importance. With the development of nanotechnology, many materials have been considered for biomedical applications and disease treatment. It is still a difficult problem to find a suitable carrier and method. It is worth noting that many traditional Chinese medicine ingredients can also be incorporated into the use of nanotechnology, which can make great progress in the follow-up research.

Nanotechnology plays an important role in the field of nanomedicine, especially in the drug delivery system. In many nanotechnology drug delivery ways, the most convenient way is oral administration, but the physiological obstacles in the body, such as the pH change in the digestive tract environment and the enzyme degradation process in the body, limit the more play of this way. Compared with traditional drug delivery, these nanostructures have several advantages. In addition to overcoming the pharmacokinetic and pharmacodynamic limitations of many potential therapeutic molecules, they may also be used for advanced drug delivery purposes, such as targeted drug delivery, controlled release, and enhanced permeability and retention (EPR) effects [86].

The challenges of nanotechnology in medical application are mainly manifested in an unstable preparation process and lack of biosafety evaluation system [25]. Due to the size limitation of nanoparticles on the nanoscale, nanoparticles can be successfully used as carriers of important therapeutic agents. The research related to the structural modification of these nanocarriers will help to avoid these problems. Oral administration of various nanopreparations can effectively reduce the adverse reactions of different diseases. Oral nanostructures have applications not only in drug delivery but also in gene therapy and vaccination. It is necessary to carry out extensive research to improve and modify the oral nanopreparations to make them more effective in application.

Although nanotechnology has been initially applied in various disease trials, a series of problems such as biodegradability, biocompatibility, drug release time, stability and integrity of biomacromolecules, and targeting of nanoparticles still need to be further improved and improved. Moreover, it is necessary to use nanotechnology in clinical treatment. Further molecular studies are needed to check its safety, in order to better serve the clinical disease treatment.

## Figures and Tables

**Table 1:** Common complications of diabetes mellitus and application of nanotechnology.

Author and year of literature	Complication	Application of nanotechnology	Functional route	Advantage	Disadvantage or notes
Yang, 2019 [46]	Diabetic nephropathy	Formulation of crocetin-loaded PLGA nanoparticles	Downregulated the production and expression of fibrotic factors viz., TGF-*β*1 and fibronectin and inflammatory cytokines including MCP-1 and TNF-*α* in renal; abated NF-*κ*B expression activation and PKC activity	Antifibrosis and anti-inflammatory effects	Only for animal experiments now
Ahangarpour, 2019 [47]	Diabetic nephropathy	Myricetin solid lipid nanoparticles (SLN)	Reducing oxidative stress and increasing antioxidant enzyme levels	The form of nanoparticles can improve the drug effect	Only for animal experiments now
Ahad, 2018 [48]	Diabetic nephropathy	The nanoliposomes containing Eprosartan mesylate	Renal protection by decreasing serum creatinine, urea, lactate dehydrogenase, and total albumin	Lowering blood pressure; reducing the degree of fibrosis	Only for animal experiments now
Fangueiro, 2015 [50] Jo, 2012 [51]	Diabetic retinopathy	Silicate (SI) nanoparticles	Inhibited VEGF-induced phosphorylation of VEGFR-2 in HRMECs	Inhibition of retinal neovascularization, nontoxic	Could be considered to use in clinical treatment
Fangueiro, 2015 [50] Kim, 2011 [52]	Diabetic retinopathy	Gold nanoparticles	Suppresses VEGFR-2 autophosphorylation followed by blocking ERK 1/2 activation and suppresses VEGFR-2 signaling pathway	Effective inhibition of retinal neovascularization, nontoxic	Could be considered to use in clinical treatment
Fangueiro, 2015 [50] Jo,2012 [51]	Diabetic retinopathy	Titanium dioxide (TiO_2_) nanoparticles	Inhibited VEGFR-2/MAPK pathway, not affecting PI3K/Akt pathway	Intravitreal injection can effectively inhibit retinal neovascularization	Could be considered to use in clinical treatment
Fangueiro, 2015 [50] Gurunathan, 2009 [54]	Diabetic retinopathy	Silver nanoparticles	Targeting the activation of PI3K/Akt signaling pathways	Inhibition of VEGF induced angiogenesis	Only for animal experiments now
Luyan, 2018 [58]	Diabetic cardiomyopathy	PSS-NP	Upregulation of PI3K/Akt/eNOS/VEGFA signaling pathway	Improve heart function, regulating the ultrastructure of microvascular endothelial cells, reduce vascular endothelial injury	Only for animal experiments now; the optimal dosage needs to be determined
Zhang, 2017 [59]	Diabetic cardiomyopathy	FGFl nanoliposomes combined with UTMD	Low-frequency ultrasound combined with microbubbles	Improve myocardial function, improve the apoptosis of cardiac cells, increase myocardial blood flow	Could be considered to use in clinical treatment; new targeted therapy
Asadi, 2019 [62]	Peripheral	Nanocurcumin on the severity of sensorimotor polyneuropathy (DSPN)	Inhibits production of proinflammatory cytokines including TNF-*α* and interleukin-1 (IL-1), and also prevents synthesis of NO	Reduces mechanical, heat, and pain allergies	Applied to clinical trials; need future long-term studies with different dose
Luo, 2019 [63]	Peripheral	Nano-miR-146a-5p	Inhibits the inflammatory response and apoptosis to reduce DPN by regulating the NF-*κ*B signaling pathway	Regulates inflammatory reaction and apoptosis, protects peripheral nerve	Only for animal experiments now; other potential mechanisms still need to be explored
Gainza, 2013 [67]	Diabetic foot	rhEGF-NPS	Induced the fibroblasts to proliferate and migrate	Accelerating the proliferation of fibroblasts	Animal experiments are limited; further research on human experiments is needed
Chen, 2012 [87] Leu, 2012 [68]	Diabetic foot	AuEA	Anti-inflammation and angiogenesis modulation	Reduces RAGE expression in fibroblasts and promote wound healing	Could be considered to use in clinical treatment
Liu, 2018 [70]	Diabetic foot	CNPsGMs/hydrogel	The GMs containing CNPs were loaded into the thermos-sensitive hydrogel responding to the MMPs that usually overexpress	Accelerates wound healing and collagen formation and improves neovascularization	Could be considered to use in clinical treatment
Sonamuthu, 2020 [71]	Diabetic foot	L-carnosine	Inhibition of MMP-9 activity and growth of bacteria; anti-inflammatory, ROS-scavenging, and antioxidant	Better wound contraction	Could be considered to use in clinical treatment
Gao, 2017 [72]	Diabetic foot	The inner loaded DMOG composite nanofiber membrane	Promotes the process of wound healing, promotes the reepithelialization and vascularization of the wound	Similar to that of natural extracellular matrix; has drug-controlled release effect	Could be considered to use in clinical treatment
Tian, 2012 [74]	Segmental bone injury	nHAC	Bone defect filling; provide framework for new bone	Shorten operation time, promote fracture healing and improve source problems	Applied to clinical trials
Ren, 2019 [75]	Segmental bone injury	Adiponectin nano	Increased the ALP activity of osteoblasts; induced more collagen secretion; promoted the expression of osteogenic related genes and their corresponding proteins	Increased ALP activity, induced collagen secretion, and extracellular matrix mineralization	Only for animal experiments now
Tang, 2019 [85]	Male fertility decline	ZnONP	Regulate DNA methylation via activating NRF1 and SIRT1	Improve sperm quantity and activity, increase serum testosterone level	Only for animal experiments now; treatment of female reproductive disorders needs to be evaluated
Liu, 2017 [79]	Diabetic macroangiopathy	Nanoselenium	Reducing oxidative stress injury of injured cells	Low concentration of nanoselenium can resist oxidation	Only for animal experiments now; treatment of female reproductive disorders needs to be evaluated

## Data Availability

No data is available.
